# Catalogue of Such British Plants as Have Been Found in Any Shape Serviceable to Man, Whether in a Medicinal, Œconomical, Culinary, or Agricultural Point of View, Together with an Account of the Uses Which They Have Been Made to Answer, and an Accurate Botanical Description of Each Plant

**Published:** 1804-06-01

**Authors:** 


### *Catalogue of such British Plants as have been found in any Shape serviceable to Man, whether in a medicinal, œconomical, culinary, or agricultural Point of View, together with an Account of the Uses which they have been made to answer, and an accurate Botanical Description of each Plant.*

( Continued from our last, pp. 439—450. )

### Triandria, Digynia.

9. Calamagrostis. *C. arenaria. Arundo arenaria.*

*Ang.* Sea mat weed, marram, helme, sea reed

*Gen. Desc.* Calyx a husk of two valves, containing one flower. Blossom hairy at the base.

*Spec. Desc.* Awn-less; *panicle* spike like; *leaves,* edges rolled inwards, pointed and thorn-like at the end; whilst growing sometimes flat with green and white streaks. *Spikes* four to six inches long, three-quarters of an inch broad. *Sea shore.* Bloss. June, July.

*Use.* Planted on the sea shore, it is of much service in preserving the adjoining fertile lands; it grows only on the very driest sand on the shore, which it prevents the wind from dispersing over the adjoining fields, as happens frequently where this grass is not found. If the seeds of this plant had been sown upon the shore, many acres, now rendered useless by the sand, might have been saved from destruction. The Dutch have profited by the discovery of this circumstance.—*Linné.* On this account the extirpation of this grass was prohibited by Queen Elizabeth; and in Norfolk, according to Mr. Woodward, it is now planted on the flat coasts, to repel the sea. That gentleman observes that where it grows, a sand-hill immediately gathers round it, and he thinks that some of our sandy cliffs may have been thus formed. The manufacture of mats and ropes of this grass, is a principal article of trade at Newborough in Anglesea.—*Withering.*

10. CALAMAGROSTIS. *C. variegata, phalaris arundinacea.*

*Ang.* Painted lady grass.

*Gen. Desc. As above.*

*Spec. Desc. Panicle* large, loose strap-spear shaped, five to eight inches long, one to two in breadth, stiff and strong, varying in colour from white to pale green in the shade, in the sun to rich shades of purple and yellow, with large dark red anthers; *leaves* broad, flat, half an inch broad, and the variety cultivated in gardens beautifully striped green and white, sometimes purplish; straw simple. *Banks of rivers, ponds.* BLOSS. July.

*Use.* It is much used in some places for thatching ricks and cottages, and is more durable than straw. Cattle eat it, but as it is of a hard texture they are not fond of it. In the province of Scandia they mow it twice a year.—*Linn.* The oftener it is mown, the more acceptable it is to cattle.

11. AIRA. *A. cæspitosa.*

*Ang.* Turfy hair grass. Hassocks, rough caps, bull's faces.

*Gen. Desc.* Calyx two-valved; two-flowered, without any intervening substance between the florets.

*Spec. Desc. Petals* woolly, awned at the base; awn straight, short; *leaves* flat; *panicle* expanding, beautifully purple and silky, six or eight inches high and half as broad; trails on the ground sometimes to the length of several feet. *In moist meadows and woods.* BLOSS. June, August.

*Use.* The ashes burnt make a good manure. Its leaves are the coarsest and roughest of all the grasses growing in pasture or meadow grounds, and, unless forced by hunger, cattle will seldom touch them. It produces abundance of leaves, but few flowering straws, and has a very disagreable appearance in meadows, being apt to grow in tufts and occasion great irregularities on the surface, and occupying ground that might produce better grass. To get rid of it the land should be drained, and the tufts pared up and burnt.—*Mr. Swayne.*

12. Melica. *M. nutans. M. montana.*

*Ang.* Mountain melic.

*Gen. Desc.* Calyx two-valved; two-flowered, with a little substance on the pedicle between the florets; nectary one leaf; stamens dilated at the base.

*Spec. Desc. Petals* not fringed; *panicle* drooping, undivided, three or four inches long.

*Use.* In the isle of Rasa they make this grass into ropes for fishing nets, which are remarkable for lasting long without rotting.—*Pennant,* 1774, *p.* 297. Cows, horses, and goats eat it.

13. Festuca. *F. fluitans.*

*Ang.* Flote fescue grass.

*Gen. Desc.* Calyx two-valved; spickets oblong, roundish; husks tapering to a point or terminating in an awn.

*Spec. Desc. Panicle* branched, upright, very long, issuing from a long two-edged sheath; *spikets* nearly sitting cylindrical awnless; *blossom* awnless, mostly ten-flowered; *straw* striking root at the joints; *leaves* floating flat on the water. *In wet ditches and ponds, common.* Bloss. June, September.

*Use.* The seeds, though small, are very sweet and nourishing. They are collected in many parts of Germany and Poland, and even brought to the tables of the great, under the name of *manna seeds,* as an agreeable and nourishing food, and are esteemed a delicacy in soup and gruels, upon account of their nutritious quality and grateful flavor.—*Lightfoot, Withering.* When ground to meal, they make bread very little inferior to that in common use, of wheat. The bran, separated in preparing the meal, is given to horses for the worms, but care must be taken that they are kept from water for some hours afterwards. Geese are very fond of the seeds, and well know where to seek them.—*Withering.* It is a good grass to sow in wet meadows, of a succulent and nourishing quality, and cattle are extremely fond of it, so much so that horses and swine will run risks to get at it.

14. Avena. *A. nuda.*

*Ang.* Naked oat, pilcorn, pills.

*Gen. Desc.* Calyx two-valved; many-flowered; awn from the back of the blossom twisted.

*Spec. Desc.* Panicled; *calyx* three-flowered, shorter than the receptacle; *petals* awned on the back, third floret awnless. The seeds when ripe fall out of the husks. *Spikets* with two or three florets. Awn neither twisted nor jointed.

Panicle

Panicle five to eight inches long, lower branches subdivided. Calyx and blossom ribbed. Blossom, July.

*Use.* This is nearly as good as the cultivated oat; it serves to make gruel or oat-cake, and feeds cattle full as well.—*Withering.* It is cultivated in Cornwall, and sells there at the price of wheat.—*Ray.* It is cultivated also in the isle of Arran on the western side of Ireland.

15. Arundo. *A. phragmites. A. vallatoria, A. vulgaris.*

*Ang.* Common reed

*Gen. Desc.* Calyx two-valved; blossom awnless; surrounded with down at the base.

*Spec. Desc.* *Calyx* five-flowered; panicle spreading; *florets* four or five, smooth, but with down at the base arising from the spike-stalk. *Rivers, lakes, ditches.* Bloss. July.

*Use.* The panicles are used by the country people in Sweden to dye woollens of a green colour. The reeds are much more durable than straw for thatching; they are used to lay across wood work, as the foundation for plaster floors, and to make garden screens against cold winds.

16. Lolium. *L. temulentum.*

*Ang.* Darnel. Annual darnel grass.

*Gen. Desc.* Calyx one leaf fixed, many-flowered; spikets alternate.

*Spec. Desc.* *Spikets* awned, compressed, many-flowered; *straw* rough when stroked upwards, and *leaves* rough when stroked downwards; *spikes* four to six inches long, rough; terminating spiket with a two-leaved calyx, and the lowermost spikets have a minute inner leaf to the calyx. *Ploughed lands, mostly among barley and flax, not common.* Blossom, July, August.

*Use.* The seeds of this grass will intoxicate man, birds, and beasts; and, taken in any considerable quantity, will bring on convulsions and death.—*Lightfoot.* Mixed with bread corn, they produce but little effect, unless the bread be eaten hot; but, if malted with barley, the ale soon occasions drunkenness.—*Linné.* Made into bread with a small proportion of wheat, and eaten repeatedly, they produced vomiting, purging, violent cholics, and death. *Monthly Rev. vol.* lxvii. *p.* 559. Sheep are not fond of this grass.

17. Elymus. *E. arenarius.*

*Ang.* Sea lime grass.

*Gen. Desc.* Calyx lateral, two-valved, several together, many-flowered.

*Spec. Desc.* *Spike* upright, compact, long, woolly; *calyx*

woolly, longer than the floret; *little spikes* two together, straight, two florets, awnless; *leaves* bluish green or whitish, rolled inwards, sharp pointed. *Sea-coast in loose sand.* Blossom, July, August.

*Use.* This grass also is of much service in resisting the dispersion and spreading of the loose sand on the seashore. Probably it might be capable of being formed into *ropes,* like the *stipa tenacissima* in Spain.—*Withering.* Cows, horses, and goats eat it; sheep refuse it.

18. Triticum. *T. repens. Gramen caninum, gramen dioscorides.*

*Ang.* Dog's grass, couch grass, squich grass, quitch grass, couch wheat.

*Gen. Desc.* Calyx two-valved, solitary, mostly three-flowered; floret bluntish.

*Spec. Desc. Calyx* four-flowered, awl-shaped, tapering to a point; *leaves* flat. *There are four varieties differing in the number of flowers of the calyx and awns. See Bot. Ar.*

*Use.* The root is the *grass-root* used in medicine; it is *diuretic* and *attenuant.—Hill, Lightfoot.* Boerhaave recommends the juice of these roots, drank liberally in obstructions of the viscera, particulaly in cases of schirrous liver and jaundice. Cattle are frequently found to have schirrous livers in the winter, and they are soon cured of them, when turned out to grass in the spring. Dogs eat the leaves of this grass to excite vomiting. At Naples, the roots are collected in large quantities, and sold in the market for the food of horses; they have a sweet taste, something approaching to that of liquorice; when dried and ground into meal they have been made into bread in years of scarcity.—*Withering.* This is a most troublesome weed in arable lands, and can only be destroyed by fallowing in a dry summer; but Mr. Pitt observes, that though this is the most common kind of *squitch* in gardens, that which is chiefly prevalent in arable lands, consists of other species of grasses, as the *agrostis,* the *holcus mollis,* and the *avena elatior. Staffordsh. Rep.* Horses eat the young leaves but leave them when fully grown; cows, sheep, and goats eat them.

Triandria Enneagynia.

19. Empetrum. *E. nigrum.*

*Ang.* Black berried heath, black crow berries, crake berries.

*Gen. Desc.* Male and female flowers on separate plants;

 Calyx

Calyx three-div. bloss. three-petals; male, stamens long; female, berry with nine seeds.

*Spec. Desc.* *Stems* trailing; *bark*, scaling off, brown, inner, yellow; *bud* terminating, of five leaves; *leaves* in fours, three-square, on leaf stalks; *flowers* from the bosom of the leaves, sitting, solitary, sometimes *herm. calyx*, whitish, *petals* and *filaments* purple, *anthers* black. On the fertile plant *leaves* in fives, deep green, sitting, rolled back; *stem* redder; *petals* dark purplish red, *anthers* flesh colour, *pistil* black, *berries* brown black. *Mountains, high heaths, rocks, bogs, moors, frequent.* Bloss. April, May.

*Use.* Boiled with alum, the berries afford a fine dark purple dye. The Highlanders frequently eat the berries, as do children sometimes, but they are not a desirable fruit; and, if taken in large quantities, they occasion head ach. Grouse feed upon them. Goats eat the leaves, but are not fond of them; horses, cows, and sheep refuse them.

### Class IV. Tetandria.

### Tetandria, Monogynia.

1. Dipsacus. *D. fullonum.*

*Ang.* Teasel, manured teasel.

*Gen. Desc.* *Calyx common*, many-leaved; *proper* superior; *receptacle* chaffy.

*Spec. Desc.* *Leaves* sitting, serrated; chaff bent backwards; scales very hard.

*Use.* This plant is cultivated for the use of clothiers, who employ the heads with crooked awns to raise the knap upon woollen cloths. For this purpose they are fixed round the circumference of a large broad wheel, which is made to turn round, and the cloth is held against them. The plant flowers in June and July, and the heads are gathered in August.—*Withering.* The leaves of this plant are a good stomachic, but they are not much known as such.—*Hill.*

2. Scabiosa. *S. succisa. Succisa glabra. Succisa sive morsus diaboli.*

*Ang.* Scabious, devil's bit scabious.

*Gen. Desc.* *Calyx common*, many-leaved; *proper*, double, superior; *receptacle* naked, chaffy; *seed* wrapped in the proper case.

*Spec. Desc.* *Blossom* four-cleft, equal; (bluish purple, flesh coloured, or white, sometimes double) *stem* undivided; *branches* approaching; *leaves* egg spear shaped; *stem and leaves* rough with hair; *flowers* in globular cups;

*proper cup* four-cornered, hairy, with four shallow clefts, the segments fringed with white hairs; *nectary* inclosing the germen, crowned with a concave glandular receptacle, armed with four or five strong reddish black bristles; each floret is besides furnished with a green spear shaped leaf, terminated by a white taper bristle. *In fields and pastures, common.* BLOSSOMS June, August.

*Use.* A strong decoction of this, continued for a good while together, is an empirical secret for *gonorrhœas.*—*Withering.* The dried leaves are used to dye wool yellow or green.—*Linné.*

3. SCABIOSA. *S. arvensis. S. pratensis hirsuta. S. major vulgaris.*

*Ang.* Common scabious. Field scabious.

*Gen. Desc.* *As above.*

*Spec. Desc.* *Blossom* four-cleft, radiating; *leaves* wing cleft, jagged, rough, spear shaped; *stem* rough with strong hairs, sometimes smooth, spotted with purple at the bottom; *flowers* sometimes white, purple, or blue, a little woolly; *proper cup* four-cornered, hairy, with four small teeth; *nectary* inclosing the germen, crowned with a concave receptacle, set with shining glands on the inside, and armed with eight or twelve spear shaped, serrated, greenish, bristly substances, hairy at the base. *Pastures and corn-fields.* BLOSSOMS July, August.

*Use.* This plant is slightly astringent, bitter, and saponaceous.—*Withering.* It is internally a pectoral, and its leaves are a common ingredient in decoctions of that kind. Externally it cures breakings out in the skin; the way of using it is boiling the juice to the consistence of ointment with lard.—*Hill.* Sheep and goats eat it, but horses and cows are not fond of it.

4. ASPERULA. *A. odorata. Rubeola montana. Aparine latifolia.*

*Ang.* Woodroof, woodrow, woodrovell, sweet woodruff, common woodruff.

*Gen. Desc.* Blossom one petal, funnel shaped; seeds two, globular, like cork.

*Spec. Desc.* *Leaves* from five to nine in a whirl, spear shaped; *flower* in bundles, on fruit stalks, of a beautiful snowy white; when magnified appears sprinkled with shining, frosted particles; calyx not evidently toothed; *fruit* covered with stiff hooked hairs; *panicle* with three divisions; from five to ten inches high. *Woods, hedges, shady places.* BLOSSOMS May.

*Use.*

*Use.* The scent of this plant is said to drive away ticks and other insects.—*Linné.* It gives a grateful flavour to wine. Horses, cows, sheep, and goats eat it.

5. Galium. *G. verum.*

*Ang.* Yellow lady's bed straw, cheese rening, petty muquet, yellow goose grass.

*Gen. Desc.* Blossom one petal, bell shaped, short; seeds two, nearly globular, beneath.

*Spec. Desc.* *Leaves* five to nine in a whirl, but generally eight on the principal stem, strap shaped, furrowed, smooth, rolled back at the edges; flower branches short; *blossom* yellow, segments greatly expanded, *styles* cloven deep; *stem* with large joints, cylindrical, scored, a little woolly. *Sides of fields and roads, frequent.* Bloss. July, October.

*Use.* The flowers are prescribed by the French in *hysteric* and *epileptic* cases. They will coagulate boiling milk. Boiled in alum water they tinge wool yellow. The roots dye a very fine red, not inferior to madder, and are used for this purpose in the island of Jura.—*Pennant.* Sheep and goats eat it, cows are not fond of it, horses and swine refuse it. This plant is subject to a disease, in which the stem and branches are set with fleshy balls, about the size of a pea, hollow within, and covered with a purplish skin.

6. Galium. *G. boreale.*

*Ang.* Cross wort madder. Cross leaved goose grass.

*Gen. Desc.* *As above.*

*Spec. Desc.* *Leaves* four in a whirl, egg spear shaped, smooth, three fibred; *stem* upright; *blossom* numerous, beautifully white, but when dried turning to a dirty yellow. *Mountains in Westmoreland and Wales.* Bloss. July, Aug.

*Use.* The roots afford a good red dye for woollen cloths.

7. Galium. *G. aparine. Aparine palustris. A. vulgaris.*

*Ang.* Goose grass, catch weed, cleavers, clivers, cleavers goose grass.

*Gen. Desc.* *As above.*

*Spec. Desc.* *Leaves* eight or ten in a whirl, strap spear shaped, rough above, smooth underneath, edges and keel set with prickles pointing backwards; *stem* four-cornered, angles set with prickles pointing backwards, joints woolly at the base; *blossom* white. *Hedges frequent.* Bloss. May, June.

*Use.* It is said by Mayerne that three ounces of the juice of this plant, taken twice a day in wine, were found an useful *aperient* and *diuretic* in incipient *dropsies*; but it

 has has been chiefly esteemed as an *antiscorbutic;* for this purpose, a tea cupful of the expressed juice is to be taken every morning for nine or ten days. When the fresh plant cannot be procured, it may be used in a dried state, as tea. Dioscorides speaks of an *ointment* made of the bruised herb, mixed with lard, as an useful application to discuss *strumous* swellings. A decoction of the plant is also said by him to have been found useful in the way of *fomentation,* in swellings of the glands of the neck, which followed a certain epidemic at Verona.—*M. M. L.* 3, *c.* 104. Dr. Cullen, however, says he has tried it in glandular indurations, without any advantage.—*M. M. ii.* 37.—*See also Med and Phil. Com. v.* 326. And, *Edwards's Treatise on the Goose Grass, &c.* Other species of galium have also been used for medical purposes, especially in *hysteric* and *epileptic* complaints.—*Woodville.* The expressed juice of the stem and leaves, taken to the amount of four ounces night and morning, is very efficacious in removing many of those cutaneous eruptions, which are called, though improperly, *scorbutic.* It must be continued for several weeks.—*Withering.* The branches are used by the Swedes for straining milk. Young geese are very fond of them. The seeds may be used instead of coffee. This plant is eaten by horses, cows, sheep, and goats; swine refuse it.—*Linné.* This plant has the effect of tinging with a florid d' colour the bones of animals that feed upon it.—*Guettard, Mem. de l'Ac. de Sc.* 1746—7.

[ To be continued. ]

